# Heat shock transcription factor 1 is SUMOylated in the activated trimeric state

**DOI:** 10.1016/j.jbc.2021.100324

**Published:** 2021-01-23

**Authors:** Szymon W. Kmiecik, Katarzyna Drzewicka, Frauke Melchior, Matthias P. Mayer

**Affiliations:** Center for Molecular Biology of Heidelberg University (ZMBH), DKFZ-ZMBH-Alliance, Heidelberg, Germany

**Keywords:** protein misfolding, stress response, transcription regulation, heat shock transcription factor 1 (Hsf1), small ubiquitin-like modifier (SUMO), post-translational modification, Aos1, SUMO-activating enzyme subunit 1, DNAJB1, DnaJ homolog subfamily B member 1, FA, formic acid, FP, fluorescence polarization, HSEs, heat shock elements, Hsf1, heat shock transcription factor 1, Hsc70, heat shock cognate 71kDa protein (HSPA8), HSR, heat shock response, PDSM, phosphorylation-dependent SUMOylation motif, PIAS, protein inhibitor of activated STAT, PVDF, polyvinylidene difluoride, RanBP2, E3 SUMO-protein ligase Ran binding protein 2, SEC, size exclusion chromatography, SUMO, small ubiquitin-like modifier, Uba2, SUMO-activating enzyme subunit 2, Ubc9, SUMO-conjugating enzyme UBC9

## Abstract

The heat shock response is a transcriptional program of organisms to counteract an imbalance in protein homeostasis. It is orchestrated in all eukaryotic cells by heat shock transcription factor 1 (Hsf1). Despite very intensive research, the intricacies of the Hsf1 activation-attenuation cycle remain elusive at a molecular level. Post-translational modifications belong to one of the key mechanisms proposed to adapt the Hsf1 activity to the needs of individual cells, and phosphorylation of Hsf1 at multiple sites has attracted much attention. According to cell biological and proteomics data, Hsf1 is also modified by small ubiquitin-like modifier (SUMO) at several sites. How SUMOylation affects Hsf1 activity at a molecular level is still unclear. Here, we analyzed Hsf1 SUMOylation *in vitro* with purified components to address questions that could not be answered in cell culture models. *In vitro* Hsf1 is primarily conjugated at lysine 298 with a single SUMO, though we did detect low-level SUMOylation at other sites. Different SUMO E3 ligases such as protein inhibitor of activated STAT 4 enhanced the efficiency of *in vitro* modification but did not alter SUMO site preferences. We provide evidence that Hsf1 trimerization and phosphorylation at serines 303 and 307 increases SUMOylation efficiency, suggesting that Hsf1 is SUMOylated in its activated state. Hsf1 can be SUMOylated when DNA bound, and SUMOylation of Hsf1 does neither alter DNA-binding affinity nor affects heat shock cognate 71kDa protein (HSPA8)+DnaJ homolog subfamily B member 1-mediated monomerization of Hsf1 trimers and concomitant dislocation from DNA. We propose that SUMOylation acts at the transcription level of the heat shock response.

Protein misfolding is detrimental for cells because of not only loss of function but also gain of toxicity of some misfolded and aggregated protein species. To cope with proteotoxic stress, a highly conserved homeostatic transcriptional program, the so-called heat shock response (HSR), emerged early in cellular evolution. The main regulator of the HSR in eukaryotic cells is the heat shock transcription factor 1 (Hsf1). Like with other transcription factors, the activity of Hsf1 is controlled on many levels (1, 2). In mammals, Hsf1 is in monomer–dimer equilibrium in unstressed cells (3). Upon proteotoxic stress, Hsf1 trimers accumulate in the nucleus and bind to heat shock elements (HSEs), three to four inverted NGAAN repeats, in promoters and enhancers to drive transcription of heat shock genes (4). One of the key mechanisms of Hsf1 regulation that allows the protein to respond to changing environmental or physiological conditions is post-translational modifications (5).

Several studies focused on phosphorylation and acetylation of Hsf1 (4, 5, 6, 7, 8, 9, 10). Hsf1 phosphorylation at multiple sites is observed upon stress induction of the HSR and was considered a hallmark of Hsf1 activation (4, 11, 12, 13, 14). Albeit phosphorylation at most sites seems to downregulate Hsf1 transactivation, phosphorylation of Hsf1 at S230 promotes Hsf1 activity (7). Similar to phosphorylation, acetylation can also either upregulate or downregulate Hsf1 activity. Hsf1 acetylation at K80 was shown to reduce DNA binding of Hsf1 and was proposed to be important for attenuation of the HSR (9). Likewise, acetylation at K118 by E1A binding protein P300 was proposed to impair functionality of Hsf1 (10). In contrast, acetylation at K208 and K298 by E1A binding protein P300 reduced degradation of Hsf1 through the proteasome resulting in enhanced HSR (10).

Less studied is the post-translational modification of Hsf1 with small ubiquitin-like modifier (SUMO), although SUMOylation per se has been intensively studied in the context of transcriptional regulation (15, 16, 17) and in HSR (see later). SUMOylation is a reversible and highly dynamic modification that regulates the function of more than thousand proteins, many of which are associated with chromatin (reviewed in Refs. (18, 19, 20)). SUMO-specific conjugating enzymes (E1 activating, E2 conjugating, and E3 ligating enzymes) form an isopeptide bond between the carboxy terminus of SUMO and the ε-amino group of lysines in an ATP-dependent reaction cascade, whereas SUMO isopeptidases revert the modification by hydrolysis. Many proteins that are SUMOylated carry a short SUMOylation consensus motif, ΨKxE (where Ψ is a large hydrophobic residue and x is any residue), which is recognized by the SUMO E2 conjugating enzyme SUMO-conjugating enzyme UBC9 (Ubc9). Vertebrates express at least three SUMO proteins: SUMO2 and SUMO3 are virtually identical, frequently form chains *via* a SUMOylation consensus motif in their flexible N termini, and their modification is strongly stimulated upon stress including heat shock. SUMO1, which shares only 50% sequence identity with SUMO2/3, exists largely in conjugated form under normal growth conditions and does usually not form chains as it lacks the N-terminal SUMOylation site (18, 20, 21).

The SUMO stress response is activated upon heat stress and results in rapid conjugation of SUMO2/3 to protein substrates (16, 22, 23). This general protein SUMOylation is proposed to be an early reaction to protein misfolding, protecting partially misfolded proteins by increasing their solubility through addition of SUMO chains (24). Consistently, cells depleted for SUMO2/3 are more sensitive to heat stress (25).

The SUMO stress response also leads to massive changes in the proteome, suggesting an influence on gene expression. SUMOylated proteins are found at sites of actively transcribed inducible genes (like heat shock genes) (25, 26, 27, 28). On one side, SUMO2/3 modification upon heat stress upregulates genes connected with not only survival and growth but also cell death (26). On the other side, SUMO2/3 modification upon heat stress represses mostly genes associated with transcription, reducing the overall load on the protein quality surveillance machinery (prosurvival function). SUMOylation was proposed to inhibit transcription reinitiation, thereby sustaining polymerase II pausing (23, 25, 28, 29). In acute stress, increase in SUMO modification is correlated with the occupancy of heat shock gene promoters by protein inhibitor of activated STAT 1 (PIAS1) SUMO E3 ligase and RNA polymerase II. Thereby, SUMOylation has been proposed as a mechanism to tightly regulate heat shock genes by preventing transcriptional hyperactivation (28).

How SUMO exerts its function in transcription regulation is largely unknown. On one side, stress-induced SUMO modification on active chromatin was proposed to act indirectly by stabilizing protein complexes on DNA rather than to act on transcription directly (26). On the other side, transcription factors are frequent targets for SUMO modification (15, 16).

Interestingly, Hsf1 is very rapidly and transiently SUMOylated upon heat stress (25, 28, 30). This transient modification is linked to phosphorylation in proximity to a conventional SUMOylation consensus motif surrounding K298, a finding that led to the first description of a phosphorylation-dependent SUMOylation motif (PDSM) (30, 31). The described PDSM consists of eight residues: ΨKxExxSP, where Ψ is a large hydrophobic residue and x is any residue. Ser 303, which is part of the PDSM, and Ser 307 (Fig. 1, *A* and *B*) can both be phosphorylated in response to heat shock. S307 phosphorylation by mitogen-activated protein kinase may be required for modification of S303 by glycogen synthase kinase 3 (13), but this is a matter of debate (30). Increased transcriptional activity has been observed for the non-SUMOylatable Hsf1–K298R and Hsf1–S303A variants in a cell culture model, strongly implying that SUMOylation downregulates Hsf1 activity (31). It has been proposed that this phosphorylation-dependent SUMOylation regulates Hsf1 by reducing its transactivation capability (30).

**Figure 1:**
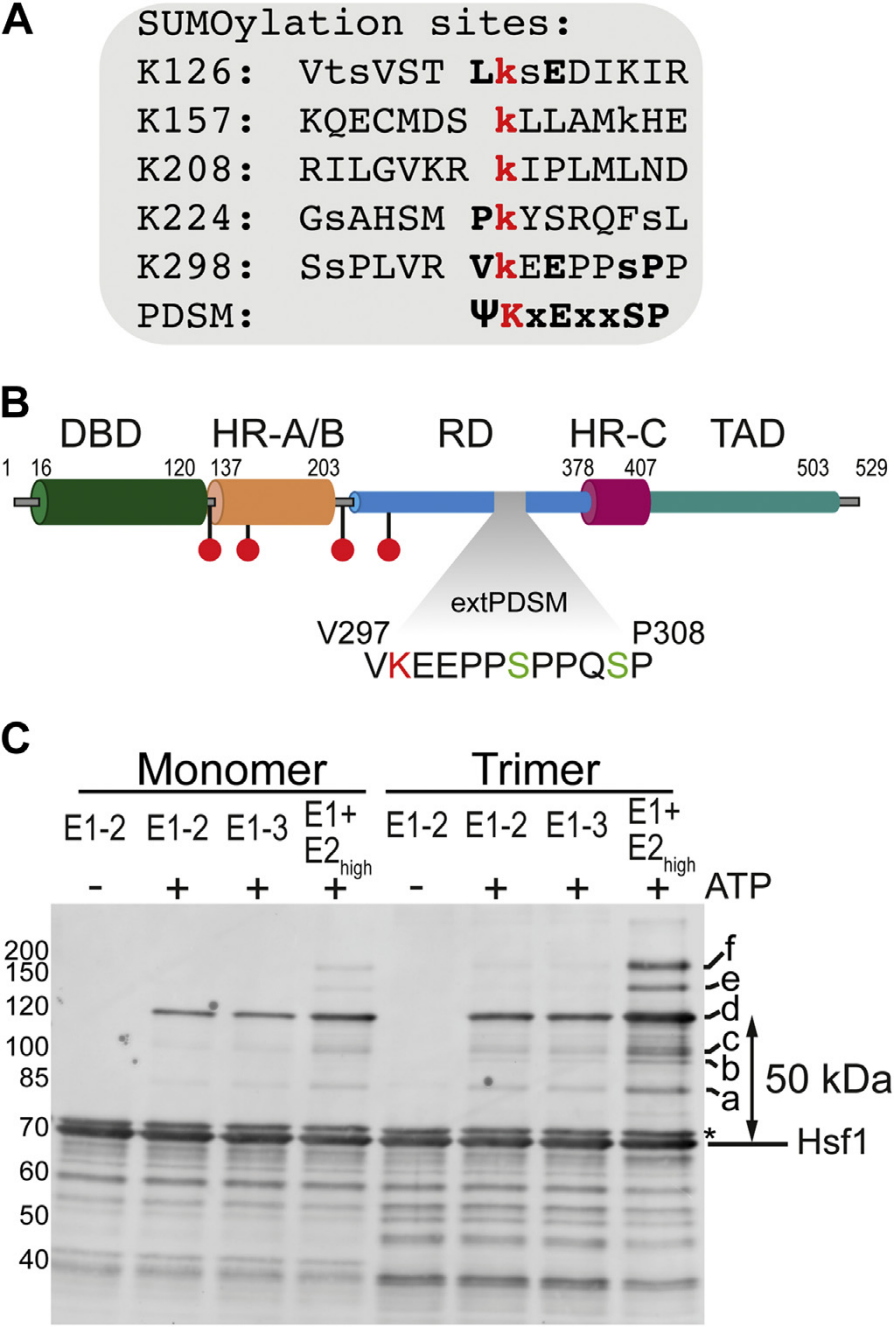
**Heat shock transcription factor 1 (Hsf1) is efficiently SUMOylated *in vitro* by E1 and E2 enzymes.***A,* SUMOylation sites within Hsf1 as identified in high-throughput MS studies. Modified lysines are indicated in *red*. Other potentially modified residues are indicated with lower cases. Residues consistent with the phosphorylation-dependent SUMOylation motif (PDSM) are shown in *bold*. *B,* Hsf1 domain organization. The extended phosphorylation-dependent SUMOylation motif (extPDSM) is indicated. Phosphorylated serines (S303 and S307) are indicated in *green*, and SUMOylated lysine residue (K298) is indicated in *red*. The position of other lysine residues reported to be SUMOylated is indicated (*red*). DBD, N-terminal winged helix-turn-helix DNA-binding domain; HR-A/B, heptad repeat trimerization domain; HR-C, third heptad repeat region important for repressing trimerization and thermosensing; RD, regulatory domain; TAD, C-terminal transactivation domain. Cylinders of smaller diameter indicate intrinsically unstructured regions within Hsf1. *C,* scanning for the best SUMOylation conditions for Hsf1 trimer and monomer. Monomeric and trimeric Hsf1–S303E,S307E were incubated with N-His–SUMO1 (10 μM), Aos1/Uba2 E1 enzyme (0.1 μM), Ubc9 E2 (low: 0.25 μM or high: 1.1 μM) in the presence and the absence of glutathione-*S*-transferase (GST)–IR1+M, a GST fusion to a fragment of the E3 SUMO ligase RanBP2 that can stimulate SUMO transfer (0.05 μM) and ATP (5 mM) as indicated, incubated for 3 h at 25 °C and subsequently separated by SDS-PAGE, blotted onto a polyvinylidene difluoride membrane and detected with an Hsf1-specific antiserum. Molecular weights in kilodalton are indicated on the *left*. ∗Contamination of purified Hsf1 with *Escherichia coli* DnaK that is also recognized by the polyclonal antiserum. SUMO, small ubiquitin-like modifier.

Whether K298 is the only relevant SUMOylation site in Hsf1, however, is not clear. Proteomics identified four additional SUMOylation sites in Hsf1 (K126, K157, K208, and K224) (Fig. 1*B*) (32, 33). One of these sites (K208) is within the sequence essential for monomerization and dislocation of Hsf1 from DNA by the Hsp70 machinery (34) and may affect this process. Consistent with the possibility that Hsf1 may carry more than one SUMO is an unusual low electrophoretic mobility of SUMOylated Hsf1 in SDS-PAGE (30).

Moreover, how SUMOylation regulates Hsf1 at a molecular level remained unclear in the published cell culture–based studies. Whether Hsf1 can be SUMOylated before activation in the monomeric state or subsequent to activation, in the trimeric state, and how exactly this modification influences Hsf1–DNA binding also remained unknown. The role of phosphorylation in regulating Hsf1 SUMOylation was also not clear. To address these questions, we reconstituted Hsf1 SUMOylation *in vitro* with purified components. We demonstrated that Hsf1 can be SUMOylated *in vitro* with high efficiency. Consistent with cell culture studies, our data indicate that lysine 298 is the preferred SUMOylation site within Hsf1 *in vitro*. Furthermore, we show that most Hsf1 molecules are conjugated with a single SUMO, and we only find traces of double SUMOylated species, suggesting a low degree of SUMO chain formation or additional SUMOylation sites *in vitro*. MS and biochemical data strongly imply that Hsf1 mono SUMOylation results in a 50-kDa upshift observed on SDS-PAGE, most likely because of branched conjugate formation. In our *in vitro* system, trimeric Hsf1 is more efficiently SUMOylated than monomeric Hsf1 by Ubc9. The phosphomimetic Hsf1–S303E,S307E variant is slightly better SUMOylated *in vitro* than wildtype Hsf1, indicating that Hsf1 phosphorylation at S303/307 is not strictly required for SUMO transfer onto Hsf1. SUMOylation did not interfere with Hsf1–DNA binding or heat shock cognate 71kDa protein (HSPA8) (Hsc70)-mediated dissociation of Hsf1 from DNA. Thus, we propose that Hsf1 SUMOylation attenuates Hsf1 action by interfering with the process of transcription itself, for example, by recruiting corepressors to heat shock gene promoters or by impairing the interaction of Hsf1 transactivation domain with the transcription machinery.

## Results

### *In vitro* Hsf1 is predominantly mono-SUMOylated at a single site

To clarify the nature of Hsf1 SUMOylation, we turned to *in vitro* SUMOylation experiments with full-length Hsf1. In light of the observation that heat stress–induced Hsf1 SUMOylation requires phosphorylation, we decided to test both wildtype Hsf1 and a phosphomimetic variant. As serines 303 and 307 are both phosphorylated under stress conditions, we considered the double phosphomimetic variant Hsf1–S303E,S307E to best imitate the *in vivo* situation.

In a first step, the conditions for Hsf1 SUMOylation *in vitro* had to be established*.* The SUMOylation reaction is catalyzed by a system of three enzymes: the E1 SUMO activating enzyme (heterodimer SUMO-activating enzyme subunit 1 [Aos1]/SUMO-activating enzyme subunit 2 [Uba2]), the E2 conjugating enzyme (Ubc9), and an E3 ligase such as one of several PIAS E3 ligases or the nucleoporin and E3 ligase E3 SUMO-protein ligase Ran binding protein 2 (RanBP2). *In vitro*, a combination of E1 and E2 seems to be sufficient to transfer SUMO to the consensus site containing substrate, and E3 ligases are proposed to accelerate the reaction or drive substrate specificity (18).

Preliminary screening for Hsf1 SUMOylation conditions demonstrated that a combination of E1 (0.1 μM) enzyme with the E2 enzyme at high concentration (1.1 μM) was sufficient to modify Hsf1 *in vitro* with His-tagged SUMO1 (Fig. 1*C*). A 70 amino acid fragment of the E3 ligase RanBP2 (IR1+M) that can stimulate SUMO transfer from the E2 conjugating enzyme to many different targets (35) did not increase efficiency of Hsf1 conjugation under these conditions (possibly because of the His tag; see below Fig. 4).

**Figure 2:**
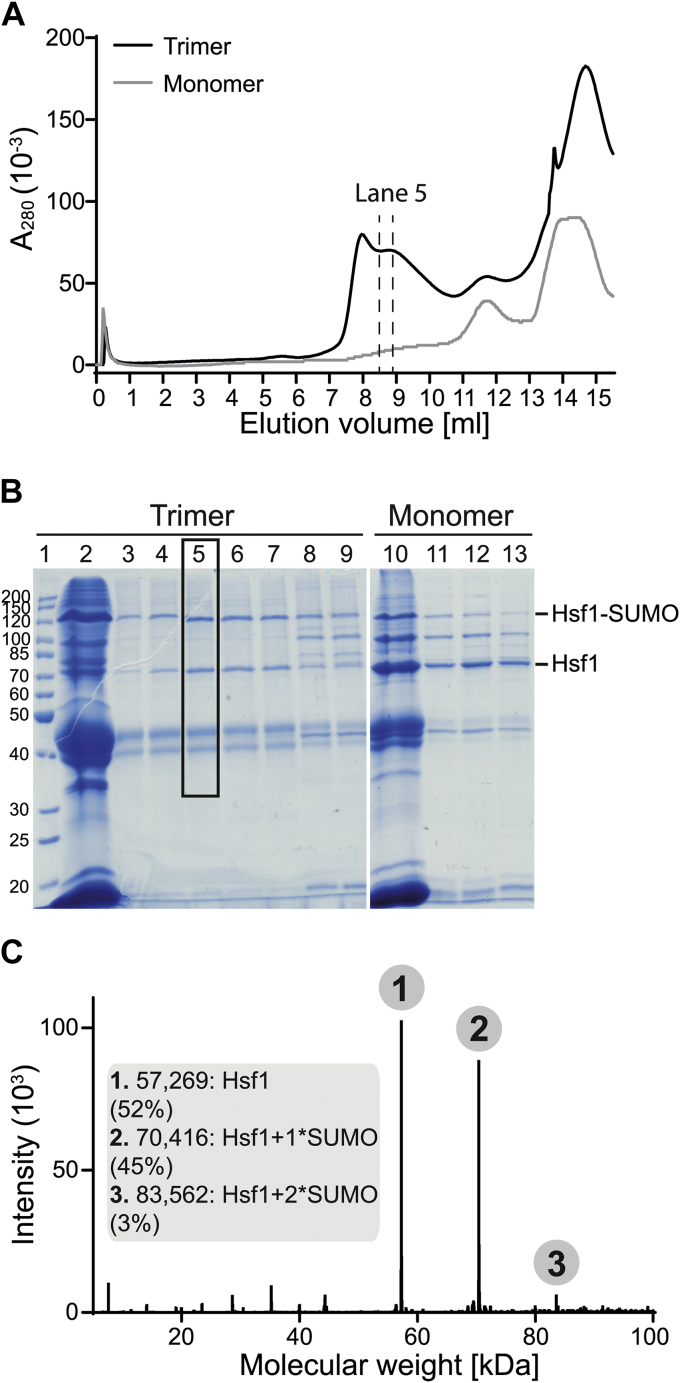
**Heat shock transcription factor 1 (Hsf1) is mostly mono-SUMOylated *in vitro****. A,* size exclusion chromatography elution profile of the SUMOylation reaction for trimeric and monomeric Hsf1–S303E,S307E. Elution only to 15.5 ml is shown for clarity. Fraction analyzed by MS (trimeric Hsf1 preparation) is indicated by *dashed lines*. *B,* SDS-PAGE analyses of collected fractions are the following: trimer: lane 1, protein ladder; lane 2, load; lanes 3 to 7, 0.5 ml fractions 7.42 to 9.92 ml; lanes 8 and 9: 0.5 ml fractions 10.92 to 11.92 ml. Fraction analyzed by MS is indicated with the frame; monomer: lane 10, load and lanes 11 to 13, 0.5 ml fractions 10.92 to 12.42 ml. Molecular weights in kilodalton are indicated on the *left*. *C,* deconvoluted MS spectrum of the analyzed fraction (lane 5) corresponding to the mixture of SUMOylated and non-SUMOylated Hsf1–S303E,S307E. Detected molecular weights together with abundance percentage are indicated in the *bracket*. N-His–SUMO1 was used in this experiment. Theoretical mass of Hsf1–S303E,S307E alone is 57,270.43 Da (measured 57,269.30 Da). Theoretical mass of N-His–SUMO1 alone is 13,164.66 Da (measured 13,164.33 Da). Theoretical mass of mono-SUMOylated Hsf1 equals 70,417.08 Da and for double-SUMOylated 83,563.73 Da. SUMO, small ubiquitin-like modifier.

Under optimal conditions, we observed six major bands above unmodified Hsf1 (labeled a to f) that are only visible in the presence of the SUMOylation machinery and ATP but not in the absence of ATP, with the dominant band (d) approximately 50 kDa above unmodified Hsf1. The N-His-SUMO1 that was used in these initial experiments has a theoretical mass of 13164.66 Da (measured 13164.33 Da), but according to previous reports, mono-SUMOylation causes an increase in apparent molecular weight on SDS-PAGE of 15 to 20 kDa (36). Therefore, we were intrigued by the fact that the molecular weight shift for the dominant band (d) above unmodified Hsf1 was around 50 kDa according to SDS-PAGE, arguing against Hsf1 mono-SUMOylation (also observed in Ref. (30)). In addition, another study investigating Hsf1 SUMOylation reported upon Hsf1 SUMOylation a shift smaller than 50 kDa arousing controversies (37). Three explanations appear possible for the observed 50 kDa increase in molecular weight of modified Hsf1: (i) the branched Hsf1–SUMO conjugate has a strongly reduced electrophoretic mobility in SDS-PAGE; (ii) Hsf1 is simultaneously SUMOylated at more than one site; and (iii) SUMO chains are formed on Hsf1 that increase the molecular weight of the conjugate. To address this question, we separated the SUMOylation reaction mixtures containing either monomeric or trimeric Hsf1 by size exclusion chromatography (SEC) and analyzed the cleanest fraction with the highest ratio of SUMOylated to unmodified Hsf1 by MS. To prevent spontaneous Hsf1 trimerization of monomeric Hsf1 *in vitro* (3), in all experiments that contained monomeric Hsf1, SUMOylation was performed at 25 °C; in experiments that contained trimeric Hsf1, SUMOylation was performed at 25 °C when performed in parallel to SUMOylation of monomeric Hsf1 (for comparison) and otherwise at 30 °C to increase the yield of SUMOylated Hsf1. SEC showed SUMOylated Hsf1 in the trimeric as well as in the monomeric fractions (Fig. 2, *A* and *B*). The mass spectrum revealed that 52% of Hsf1 were unmodified, 45% were modified by a single SUMO, and less than 3% were modified by two SUMO molecules (Fig. 2*C*). Comparing these values with a quantification of the bands of the immunoblot indicates that the modification with a single SUMO can cause a shift of up to 50 kDa in SDS-PAGE (Figs. 1*C* and 2*C*). This indicates a much stronger context dependency of the electrophoretic mobility shift induced by SUMOylation, than what is generally believed. The bands (a–c) between the band of unmodified Hsf1 and the major SUMOylation band (d) are likely to also represent mono-SUMOylated Hsf1, albeit at different sites that cause a smaller electrophoretic mobility shift. In line with this, the phosphosite database (www.phosphosite.org; October 2020) lists five Hsf1 lysines that can be SUMOylated: K126, K157, K208, K224, and K298 (Fig. 1, *A* and *B*). The much fainter bands between c and d might contain SUMOylated truncated versions of Hsf1, as immunoblotting of purified Hsf1 also contained bands below the main Hsf1 band that presumable contain Hsf1 variants truncated within the C-terminal unstructured transactivation domain by contaminating proteases during purification. These bands also disappeared during the SUMOylation reaction, presumably upshifted upon SUMOylation. The bands above the major SUMOylation band (bands e and f) then most likely represent modifications with two SUMO moieties (at different sites or formation of SUMO chains). In these preliminary experiments (Figs. 1 and 2), N-terminally histidine-tagged SUMO1 was used in the SUMOylation reactions; however, as additional amino acids introduced to a protein may change its properties, untagged versions of SUMO1 and SUMO2 were used in all further experiments.

### K298 is the primary Hsf1 SUMOylation site, and its SUMOylation *in vitro* does not require phosphorylation

**Figure 3:**
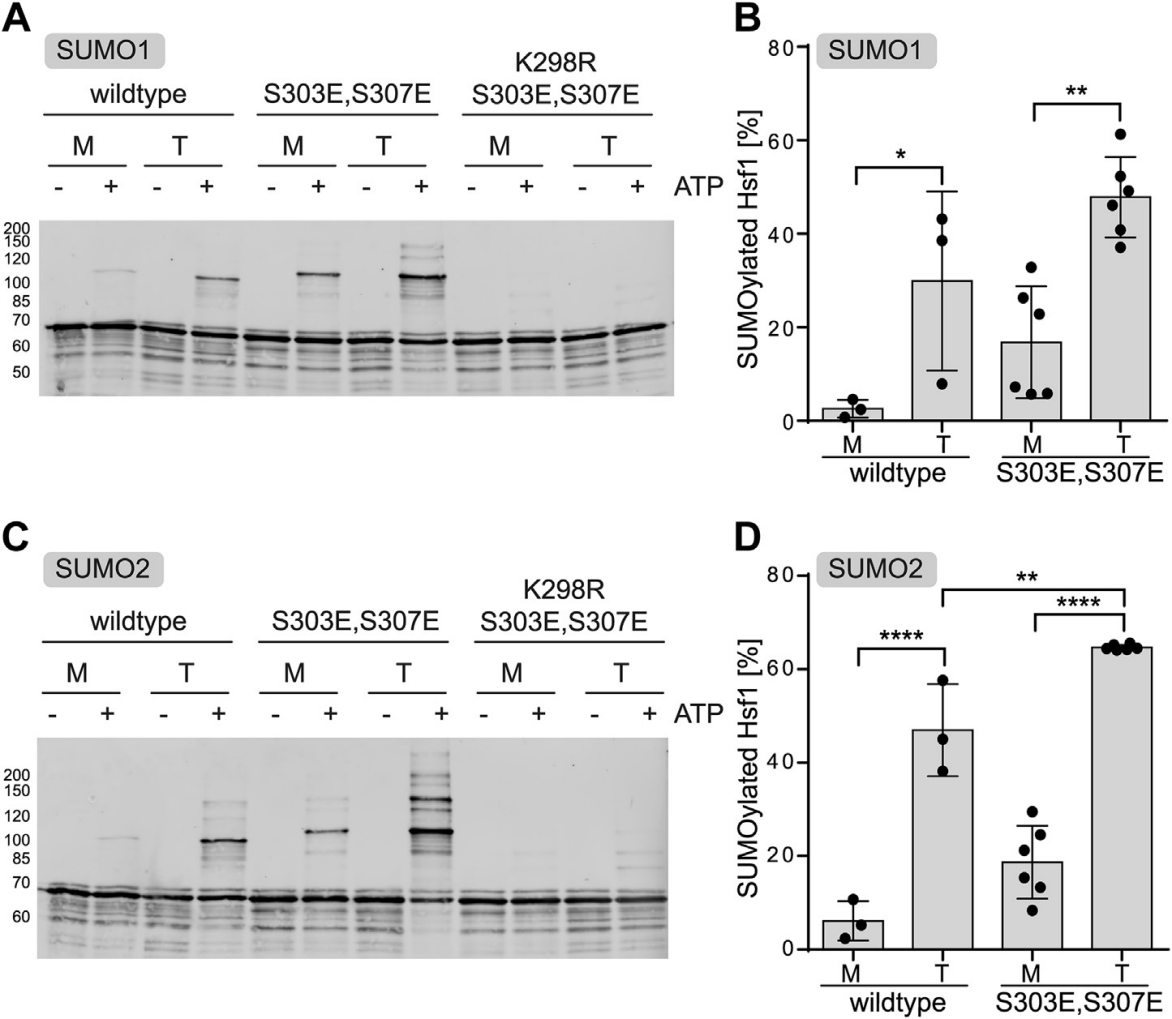
**Lysine K298 is the primary SUMOylation site within heat shock transcription factor 1 (Hsf1).***A,* comparison of SUMOylation efficiency for monomeric and trimeric Hsf1 wildtype Hsf1–S303E,S307E and Hsf1–K298R,S303E,S307E with SUMO1. Monomeric Hsf1 was heat shocked for 10 min at 42 °C to convert it into trimers. Subsequently, monomeric and trimeric Hsf1 were incubated for 3 h at 25 °C with SUMO1, E1 and E2 enzymes plus or minus ATP as indicated, then separated by SDS-PAGE, blotted onto a PVDF membrane and detected with an Hsf1-specific antiserum. A representative Western blot is shown. Molecular weights in kilodalton are indicated on the *left*. *B,* quantification of SUMOylation results from panel *A*. The percentage of monomodified conjugate was calculated. Shown are mean ± SD (wildtype n = 3, S303E,S307E n = 6, ANOVA, ∗*p* < 0.05; ∗∗*p* < 0.01). *C,* comparison of *in vitro* SUMOylation efficiency for wildtype Hsf1, Hsf1–S303E,S307E and Hsf1–K298R,S303E,S307E with SUMO2. Samples were prepared as described for panel *A*, separated by SDS-PAGE, blotted onto a PVDF membrane and detected with an Hsf1-specific antiserum. A representative Western blot is shown. Molecular weights in kilodalton are indicated on the *left*. *D,* quantification of SUMOylation results from panel *C*. The percentage of monomodified conjugate was calculated. Shown are mean ± SD (wildtype n = 3, S303E,S307E n = 6, ANOVA, ∗∗*p* < 0.01; ∗∗∗∗*p* < 0.0001). SUMO, small ubiquitin-like modifier.

**Figure 4:**
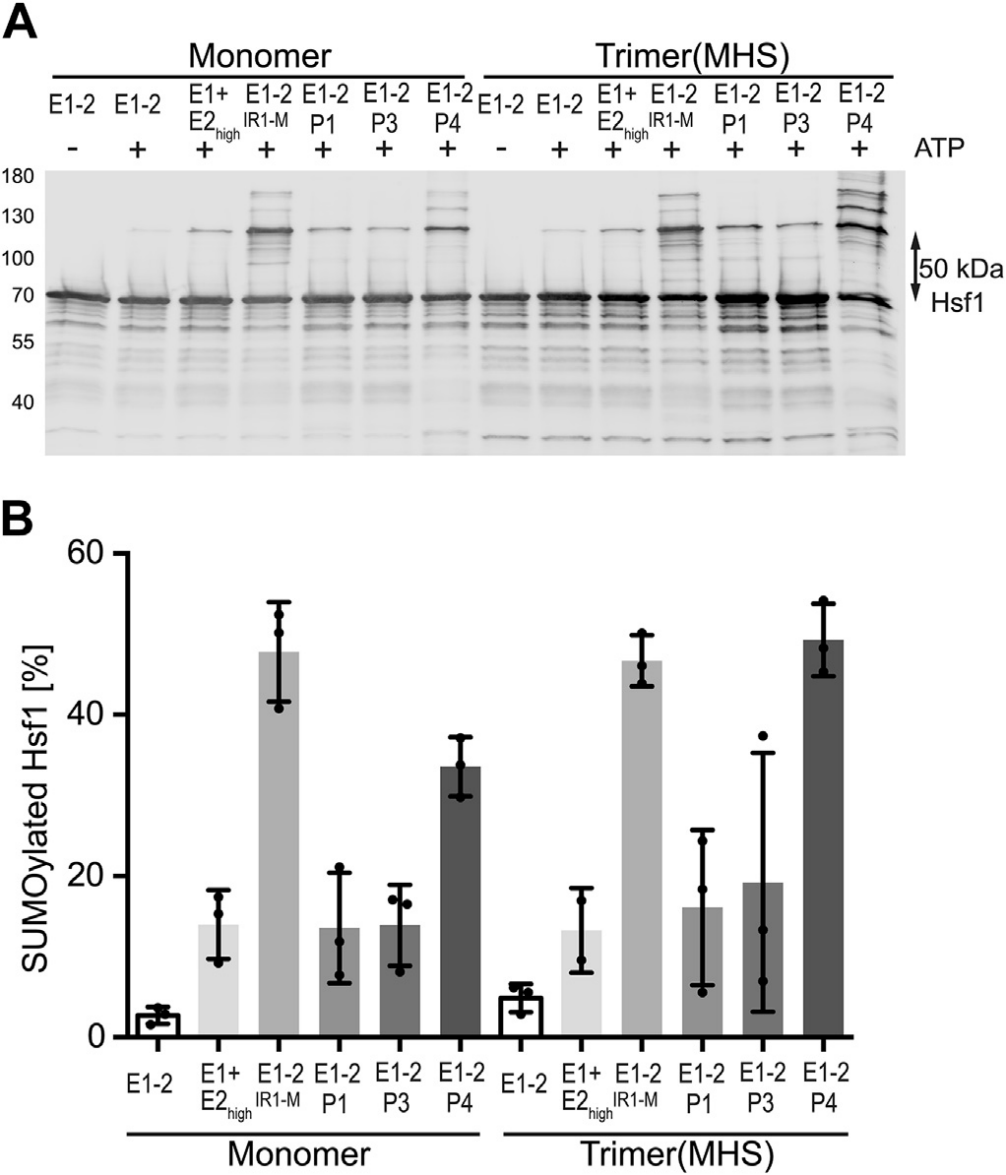
**Heat shock transcription factor 1 (Hsf1) SUMOylation is enhanced by E3 SUMO ligases RanBP2–IR1+M, PIAS1, PIAS3, and PIAS4**. *A,* monomeric and trimeric (monomer heat shocked at 42 °C for 10 min) Hsf1–S303E,S307E were incubated with SUMO2 (10 μM), Aos1/Uba2 E1 enzyme (0.1 μM), Ubc9 E2 (low: 0.25 μM or high: 1.1 μM) in the presence and absence of the E3 SUMO ligase domain IR1+M of RanBP2, GST–PIAS1, GST–PIAS4 (0.05 μM), and GST–PIAS3 (0.024 μM) and ATP (5 mM) as indicated for 1 h at 25 °C and separated by SDS-PAGE followed by immunoblotting with Hsf1-specific polyclonal antiserum. *B,* quantification of three independent experiments as shown in panel *A*. Shown are mean ± SD and individual data points. SUMO, small ubiquitin-like modifier.

Demonstrating that Hsf1 can be mainly mono-SUMOylated, we wondered whether K298 is the major SUMOylation site within Hsf1 in our *in vitro* system*.* To evaluate this hypothesis *in vitro*, K298 was replaced by arginine in the double phosphomimetic Hsf1–S303E,S307E variant. Consistent with the published cell culture data, the major band (d) of SUMO–Hsf1 almost completely disappeared upon replacement of K298 with arginine, but the bands a and c were still visible, arguing that these bands represent Hsf1 species that are SUMOylated at any of the other sites found by proteomics (Fig. 3, *A* and *C*). The experiments discussed previously were done using a minimal SUMOylation setup that included only the E1 and a high concentration of the E2 enzyme. We thus wondered whether Hsf1 SUMOylation pattern would be similar in the presence of PIAS E3 ligases that are enriched in the nucleus and which have been observed on chromatin. As shown in Figure 4, this is indeed the case—irrespective of whether PIAS1, PIAS3, or PIAS4 was used, the pattern of SUMOylated Hsf1 bands remained similar, with a dominant band at approximately 120 kDa, indicative of Hsf1 K298 SUMOylation. Of note, in this experimental setting, which involved untagged SUMO2, the RanBP2 fragment IR1+M had the expected stimulatory effect, and again the major product of the SUMOylation reaction exhibited an apparent 50 kDa size shift.

Phosphorylation on S303 was proposed to be an essential prerequisite for Hsf1 SUMOylation in cells (30). This is not the case *in vitro* where also wildtype Hsf1 can be SUMOylated with SUMO1 and SUMO2 albeit with lower yields than the phosphomimetic protein (Fig. 3*A–D*).

### Trimeric Hsf1 is more efficiently SUMOylated than monomeric Hsf1

Upon heat shock, Hsf1 shifts from a monomer–dimer equilibrium to the trimeric state upon which it acquires DNA-binding competency and releases paused RNA polymerase for transcription of heat shock genes. The HSR is attenuated by Hsp70-mediated monomerization and dissociation of Hsf1 from DNA. Evidence was provided that SUMOylation attenuates Hsf1 activity. There are several possible mechanisms how SUMOylation could influence Hsf1-driven heat shock gene transcription. (i) SUMOylation could prevent Hsf1 trimerization, (ii) SUMOylation could interfere with binding of Hsf1 to its recognition sequence (HSEs) in promoter DNA or cause spontaneous dissociation from DNA; (iii) Hsf1 SUMOylation could change the kinetics of Hsp70-mediated Hsf1 dissociation from DNA; and (iv) the interaction with the transcription machinery could be modulated, and transcription reinitiation may be inhibited after Hsf1 SUMOylation (as suggested in Refs. (28, 29)). The first scenario would imply that Hsf1 is SUMOylated in the monomeric state and that this modification slows down the transition to the trimeric DNA-binding state. We therefore wondered whether Hsf1 is preferentially SUMOylated in the monomeric state or in monomeric and trimeric states to similar extent. To address this question, we generated Hsf1 trimers *in vitro* by heat shocking monomeric Hsf1 for 10 min at 42 °C. Once formed, these trimers are stable (see later), which allowed us to directly compare them with monomeric Hsf1 for SUMOylation efficiency at 25 °C *in vitro*. In the presence of the minimal SUMOylation system consisting of E1 (Aos1/Uba2) and E2 (Ubc9) enzymes, Hsf1 trimers were SUMOylated with twofold to threefold higher efficiency than Hsf1 monomers for both SUMO1 and SUMO2 (Fig. 3). Moreover, in our *in vitro* assays, trimeric Hsf1 was more efficiently SUMOylated with SUMO2 (SMT3A) than with SUMO1 (SMT3C) (Fig. 3, *B* and *D*), consistent with the fact that upon proteotoxic stress, increased SUMO2 but not SUMO1 modifications of Hsf1 were observed in cell culture studies (25, 31).

To verify that the quaternary structure of Hsf1 did not change during the incubation time, we incubated monomeric and trimeric (Hsf1 monomers heat shocked at 42 °C for 10 min) wildtype Hsf1, Hsf1–S303E,S307E, and Hsf1–K298R,S303E,S307E for 3 h at 25 °C and compared samples taken at 0 and 3 h by SDS-PAGE and Blue native-PAGE (BN-PAGE) (Fig. S1). During the incubation period, the small trimeric fraction in the monomeric Hsf1 stayed roughly the same, and no monomeric fraction appeared in the samples containing trimeric Hsf1. Therefore, there is very little transition from monomer to trimer and no spontaneous dissociation of trimeric Hsf1.

### SUMOylation of Hsf1 does not affect the monomer–trimer transition

**Figure 5:**
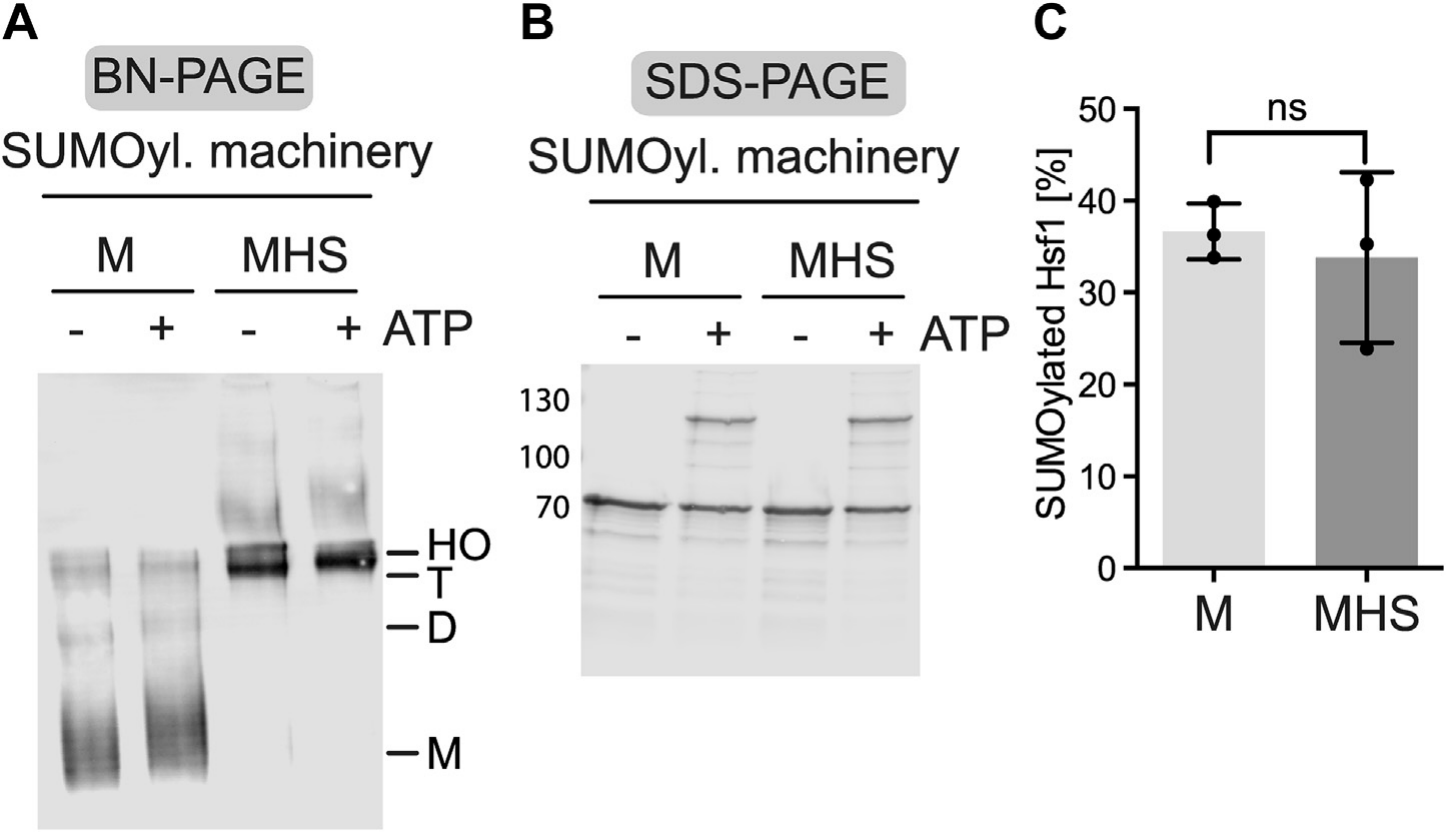
**SUMOylation of heat shock transcription factor 1 (Hsf1) does not affect heat induced monomer-trimer transition.***A* and *B*, Hsf1–S303E,S307E (M, monomer; MHS, monomer heat shocked at 42 °C for 10 min) were incubated with the SUMOylation machinery (SUMO2 [10 μM], Aos1/Uba2 E1 enzyme [0.1 μM], Ubc9 E2 [0.25 μM], RanBP2–IR1-M [0.05 μM] plus or minus ATP [5 mM]) for 1 h at 25 °C. Subsequently, the samples were split, and one aliquot incubated at 4 °C, whereas the other aliquot was heated at 42 °C for 10 min. All samples were analyzed by BN-PAGE (*A*) and SDS-PAGE (*B*) with subsequent immunoblotting using Hsf1 specific antisera. *C,* quantification of the relative amount of SUMOylated Hsf1 as shown in (*B*) and two similar immunoblots. SUMO, small ubiquitin-like modifier.

As described previously, Hsf1 can be SUMOylated both in the monomeric state but even better in the trimeric state. We next wanted to test whether SUMOylation would affect the monomer–trimer transition. We therefore incubated monomeric Hsf1 in the presence of the SUMOylation machinery with or without ATP for 1 h at 25 °C and subsequently heat shocked a fraction of each sample at 42 °C for 10 min to convert monomeric Hsf1 into trimers. Analysis of the samples by BN-PAGE (Fig. 5*A*) and SDS-PAGE (Fig. 5, *B* and *C*) revealed that Hsf1 was SUMOylated in the presence of ATP but not in its absence and that both samples trimerized with equal efficiency upon heat shock. If SUMOylation would inhibit trimerization, we should have observed some remaining monomer in the sample that contained SUMOylated Hsf1, which clearly is not the case, precluding the first hypothesis as reason for the inhibitory effect of SUMOylation on Hsf1 activity.

### Hsf1 SUMOylation and Hsf1–DNA binding are independent and do not influence each other

**Figure 6:**
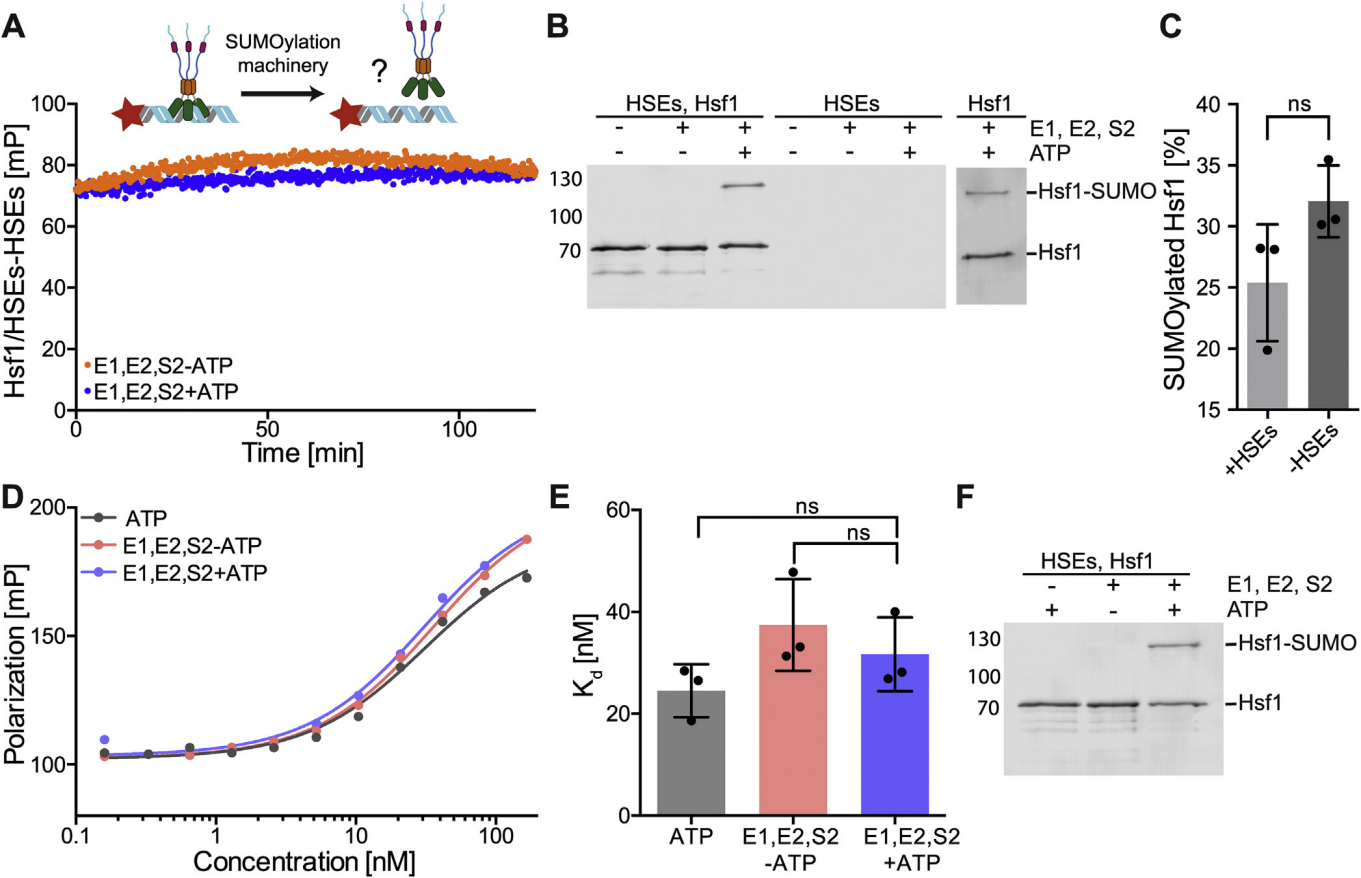
**Heat shock transcription factor 1 (Hsf1) SUMOylation does not affect DNA binding.***A,* SUMOylation of DNA-bound trimeric Hsf1 monitored by fluorescence polarization (FP) as indicated by the cartoon above the graph. Hsf1–S303E,S307E was bound to fluorescent-labeled HSE–DNA, then the SUMOylation machinery plus or minus ATP was added, and FP was monitored. Plotted are values corrected for control samples where Hsf1 was absent. *B,* Western blot of samples analyzed in panel *A*. In addition to the right, a sample incubated in the absence of HSE–DNA with the SUMOylation machinery is shown. *C,* quantification of SUMOylation efficiency in the presence and absence of DNA (HSEs). Shown are mean ± SD (n = 3, Student's *t* test, unpaired; ns, not significant). *D,* equilibrium titration of Hsf1–S303E,S307E (SUMOylated trimeric Hsf1 *versus* non-SUMOylated samples) binding to Alexa 488–labeled HSE–DNA. FP is plotted *versus* the Hsf1–S303E,S307E trimer concentration. *E,* DNA-binding affinity for SUMOylated and non-SUMOylated Hsf1–S303E,S307E. Shown are mean ± SD (n = 3, ANOVA, Sidak's multiple comparison; ns, not significant). *F,* a representative Western blot of samples analyzed in panel *D*. About 32% of Hsf1 was SUMOylated in this experiment. SUMO, small ubiquitin-like modifier.

To address the second possibility, we bound Hsf1 to fluorescently labeled HSEs containing DNA and subsequently added the SUMOylation machinery (Fig. 6*A*). Under these conditions, about 25% of Hsf1 was SUMOylated (Fig. 6, *B* and *C*). However, we did not observe any significant decrease in fluorescence polarization (FP) during the SUMOylation reaction as compared with a reaction in the absence of ATP, which precludes SUMOylation, indicating that although Hsf1 is SUMOylated, it can still stay bound to DNA (HSEs). Moreover, an equilibrium titration to investigate the affinity of SUMOylated Hsf1 to DNA did not reveal any significant difference in *K*_*D*_ values (Fig. 6, *D–F* indicates SUMOylation status of the analyzed samples). Consistent with Sistonen *et al*. (30), our data demonstrate that binding of Hsf1 to DNA does neither inhibit nor stimulate the SUMOylation process, implying that SUMOylation may occur after Hsf1 binding to DNA (Fig. 6, *B* and *C*). This observation, however, is not consistent with the results of Hong *et al*. (37) showing that incubation of Hsf1, produced in reticulocyte lysate, with a semipurified SUMO1 modification system increased binding of Hsf1wt but not Hsf1–K298R to DNA in an EMSA. Albeit, in this published experiment, neither Hsf1 amounts nor SUMOylation of Hsf1wt was verified by immunoblotting.

**Figure 7:**
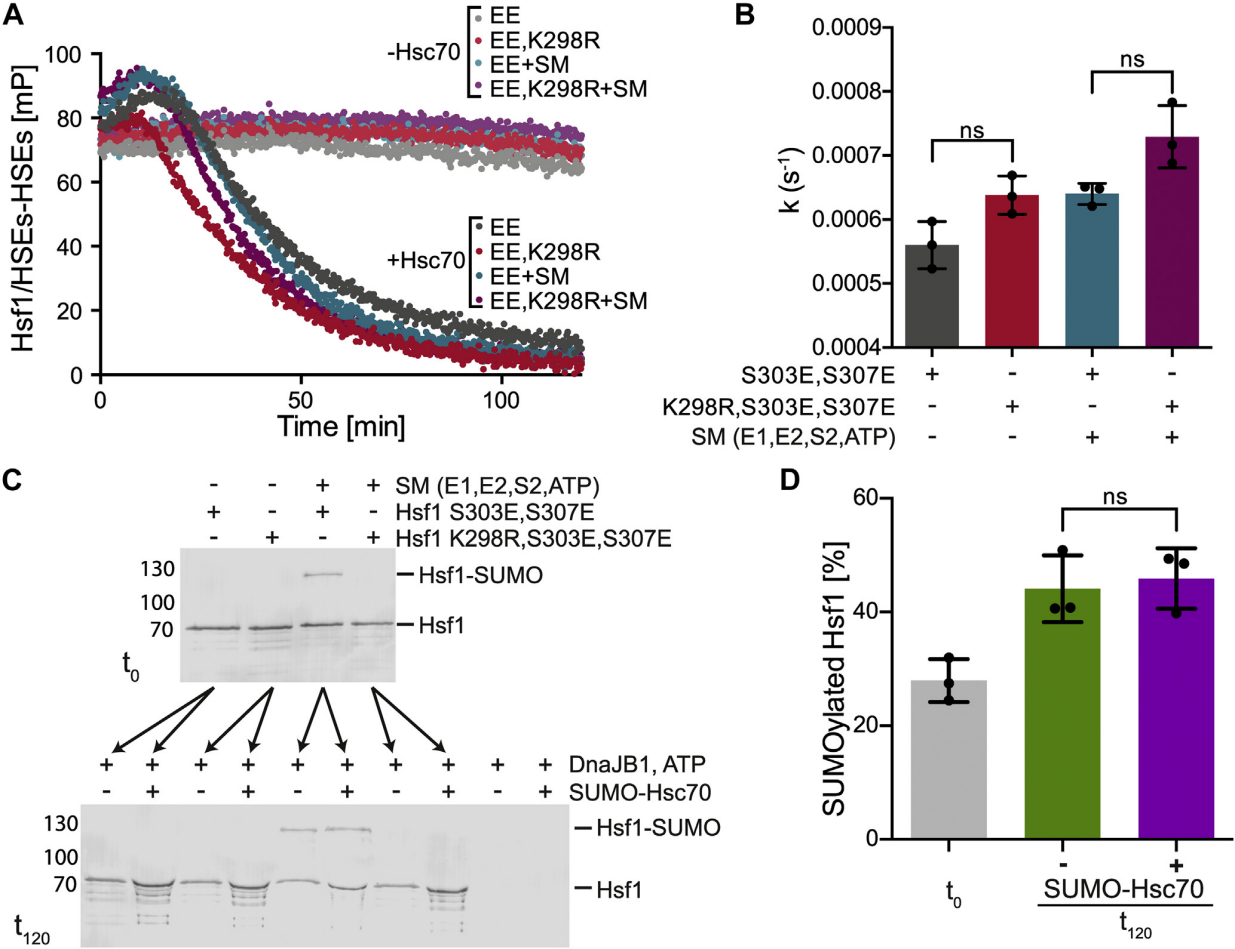
**SUMOylation of heat shock transcription factor 1 (Hsf1) does not influence Hsc70+DnaJB1–mediated dissociation of Hsf1 from DNA.***A,* dissociation of SUMOylated and non-SUMOylated Hsf1 from DNA (HSEs) by the Hsp70 system (SUMO–Hsc70, DnaJB1/Hdj1, ATP). Hsf1–S303E,S307E (EE) and Hsf1–K298R,S303E,S307E (EE,K298R) were incubated in the absence or presence of the SUMOylation machinery (SM; E1 + E2 + SUMO2 + ATP) for 1 h at 30 °C, then bound to fluorescent-labeled HSE–DNA and subsequently incubated with (+Hsc70) or without (−Hsc70) SUMO–Hsc70 + DnaJB1/Hdj1 + ATP while monitoring fluorescence polarization. A representative graph is shown. *B,* comparison of dissociation kinetics for different Hsf1 variants (SUMOylated Hsf1–S303E,S307E and non-SUMOylable Hsf1–K298R,S303E,S307E in the presence and absence of the SUMOylation machinery). Shown are mean ± SD (n = 3, ANOVA, Sidak's multiple comparison; ns, not significant). *C,* representative Western blot of samples before (*t*_0_) and after (*t*_120_) the dissociation kinetics measurement. *D,* comparison of SUMOylation efficiency before dissociation reaction and after the reaction (sample in the presence and absence of SUMO–Hsc70). Shown are mean ± SD (n = 3, ANOVA; ns, not significant). SUMO, small ubiquitin-like modifier.

Using the previously established FP assay, the influence of SUMOylation on Hsp70-mediated Hsf1 dissociation from fluorescently labeled HSEs containing DNA was evaluated. In short, both heat-inducible Hsp70 and constitutive Hsc70 in cooperation with their cochaperone DnaJ homolog subfamily B member 1 (DnaJB1)/Hdj1 are able to dissociate Hsf1 from DNA through ATP hydrolysis–driven cycles of entropic pulling, unzipping the triple leucine zipper of the trimer interface of Hsf1, thereby decreasing FP of the labeled HSE–DNA (34). To test the influence of SUMOylation on this process, trimeric Hsf1–S303E,S307E (EE) and Hsf1–K298R,S303E,S307E (REE) were incubated in the presence or the absence of the SUMOylation machinery for 1 h at 30 °C, bound to HSE–DNA and subsequently subjected to Hsc70-mediated dissociation from DNA. In the absence of Hsc70, no changes in FP were observed indicating that all Hsf1 variants remained bound to DNA as expected (Fig. 7*A*). In the presence of Hsc70, FP decreased with time for all samples independent of the Hsf1 variant (SUMOylatable or not) used or the absence or the presence of the SUMOylation machinery. No significant differences in the kinetics of Hsc70-driven dissociation from DNA could be detected for the SUMOylated double phosphomimetic Hsf1 variant in comparison to the non-SUMOylatable Hsf1–K298R,S303E,S307E variant (Fig. 7). Previously, we observed differences in dissociation kinetics when using heterotrimers of Hsf1wt and an Hsf1 variant that cannot be dissociated by Hsc70 from DNA because of the missing Hsc70 binding site, formed by mixing the respective monomers at different wildtype:mutant ratios (including 2:1) and heating at 42 °C for 10 min (34). Therefore, the applied dissociation assay should be sensitive enough to detect differences in dissociation kinetics, if the SUMOylated Hsf1 fraction (28%, calculated in Fig. 7, *C* and *D* for time point 0 and 44 and 46% after 2 h) could not be dissociated from the DNA by the Hsp70 system. Moreover, Hsf1 SUMOylation did not impair the interaction with Hsc70 implying that *in vivo* Hsf1 de-SUMOylation by SUMO isopeptidases could occur not only while Hsf1 is bound to DNA but also after Hsc70-mediated dissociation from DNA.

## Discussion

In this study, we answered several questions that could only be addressed in an *in vitro*–reconstituted Hsf1 SUMOylation system with purified components. (1) We showed that in the absence of E3 ligases or in the presence of PIAS4, trimeric Hsf1 is SUMOylated more efficiently than monomeric Hsf1 suggesting that Hsf1 SUMOylation may occur after stress-induced activation. (2) Hsf1 is primarily mono-SUMOylated, and only a minor fraction carries two SUMOs. (3) Phosphomimetic Hsf1–S303E,S307E is SUMOylated only slightly better than Hsf1wt, demonstrating that SUMO conjugation does not strictly depend on prior phosphorylation. (4) SUMOylation of Hsf1 does not affect trimerization, its DNA binding, or its Hsc70+DnaJB1–mediated dissociation from DNA and monomerization.

In accordance with previous cell culture studies, lysine 298 is the major SUMOylation site within Hsf1 *in vitro*, when using only the E1 SUMO activating enzyme Aos1/Uba2 and the E2 SUMO conjugating enzyme Ubc9, and most likely also in the presence of PIAS E3 ligases. We also observe some SUMOylation at other sites in Hsf1–K298R,S303E,S307E as well as in wildtype Hsf1, consistent with proteomics studies (33). Therefore, our *in vitro* SUMOylation system mirrored the *in vivo* situation. Among the *in vitro* tested SUMO E3 ligases, PIAS4 very efficiently SUMOylated Hsf1. Published findings revealed that heat stress recruits PIAS4 (also called PIASy), Ubc9, and SUMO2 to the HSPA1A promoter that also contains binding sites (HSEs) for Hsf1 (38). It is thus quite conceivable that Hsf1 is SUMOylated when bound to heat shock promoters.

In our assays, monomeric and trimeric Hsf1 can be distinguished, which is of advantage over previous cell culture studies where this was not feasible. We demonstrate that trimerization and phosphorylation, two events accompanying Hsf1 activation, enhance Hsf1 SUMOylation *in vitro*. One possible explanation for an increased SUMOylation efficiency in trimeric Hsf1 could be the close spatial proximity of several SUMOylation sites in the trimeric state. Alternatively or in addition, a change of conformation in Hsf1 upon transition from the monomeric to the trimeric state could facilitate SUMO modification. The reason for more efficient SUMOylation of phosphorylated Hsf1 could reside in the electrostatics of the active site of Ubc9. The crystal structure of Ubc9 in complex with RanGAP1 (39) reveals a positive patch at a position from the active center where phosphorylated S303 would be in Hsf1, suggesting that phosphorylation at S303 helps to position K298 in the catalytic center of Ubc9. Nevertheless, Hsf1 wildtype protein can also be SUMOylated *in vitro*, indicating that S303 and S307 phosphorylation is not necessary for Hsf1 SUMOylation *in vitro*. This might also explain why the other potential SUMOylation sites are SUMOylated in our assay, albeit inefficiently, as they do not contain an acidic residue or a phosphorylatable serine or threonine five residues downstream of the SUMOylated lysine, a position corresponding to S303 for K298. Except for one (K126), they also do not contain a glutamate in position +2 or a large hydrophobic residue in position −1 (Fig. 1). The somewhat larger difference in SUMOylation efficiency between Hsf1wt and Hsf1–S303A in cell culture experiments as compared with the difference of unphosphorylated Hsf1wt and Hsf1–S303E,S307E *in vitro* may be due to several different effects. First, Hsf1–S303A may not be a good surrogate for unphosphorylated Hsf1, as the hydrophilic amino acid serine in position 303 may stabilize the Hsf1–Ubc9 complex more efficiently than the hydrophobic alanine. Second, Hsf1–S303E,S307E may not be a perfect surrogate for phosphorylated Hsf1, as the larger phosphate group might interact more favorably with Ubc9 than the carboxylic group in glutamate, making the difference in SUMOylation efficacy more perceptible. Third, SUMOylated Hsf1wt might be more efficiently de-SUMOylated by SUMO isopeptidases than phosphorylated Hsf1.

Hsf1 activity upon SUMOylation may be regulated by several different mechanisms. SUMO modification could compete with other modifications like acetylation or ubiquitination for target lysines (15, 21). Competition between SUMO and ubiquitin modification could lead to stabilization of the protein by preventing ubiquitin-mediated targeting to the proteasome (40, 41, 42). In such a case, SUMOylation would be expected to increase Hsf1 concentrations and seems inconsistent with the inhibitory effect of SUMOylation on Hsf1 activity. Also, ubiquitination of K298, the major site for SUMOylation, has not been reported so far, in contrast to ubiquitination of K208 that is likewise SUMOylated, albeit to much lower degree than K298. SUMOylation has been reported to affect the distribution of proteins between cytoplasm and nucleus (43). The subcellular localization of Hsf1 is currently still debated, and some publications show Hsf1 mostly in the nucleus, whereas other publications show it in the cytoplasm in unstressed cells and the nucleus after heat shock (44, 45, 46, 47). Reduced import of Hsf1 into the nucleus could explain the SUMOylation-linked attenuation of the HSR. However, we consider such a mechanism for SUMO action on Hsf1 as less likely since our data show that Hsf1 can also be efficiently SUMOylated in the trimeric state when bound to DNA. SUMOylation has been described to inhibit DNA binding of the modified transcription factor as in the case of p53 (44) but also in contrary, to stimulate DNA binding as in the case of Hsf2 that is proposed to be converted into the DNA-binding active state by modification with SUMO1 at Lys82 in the DNA-binding domain (45). Our study demonstrates that Hsf1 SUMOylation does not impair binding of this transcription factor to DNA (consistent with Refs. (31, 46)), excluding SUMOylation-dependent dissociation from DNA as mechanism for SUMOylation-induced attenuation of the HSR. In addition, binding of SUMO target proteins to DNA may regulate the modification efficiency *in vitro* and *in vivo* (proliferating cell nuclear antigen (PCNA) (47) and poly(ADP-ribose) polymerase 1 (48, 49)). This is not the case for Hsf1 *in vitro*. DNA binding did not significantly increase Hsf1 SUMOylation yields under our conditions, indicating that SUMOylation and DNA binding are two independent events, and this modification may occur before or after Hsf1 binding to DNA. In the context of more extended DNA fragments or chromatin, this might be different. Hsf1 SUMOylation also does not interfere with Hsc70-mediated dissociation of Hsf1 from DNA, which has recently been shown to be a major factor in Hsf1 activity attenuation (34). Our data suggest that Hsf1 SUMOylation may take place after Hsf1 binding to DNA (in line with Refs. (23, 28)), and subsequent Hsf1 de-SUMOylation may occur before or after Hsc70-mediated Hsf1 dissociation from DNA. To regulate transcription, Hsf1 needs to interact with a large number of factors including components of the transcriptional machinery and complexes remodeling the chromatin. Hsf1 SUMO modification can inhibit such interactions by masking an interaction interface or promote additional interactions, for example, with proteins containing a SUMO-interacting motif, changing the interactome of Hsf1 (16, 17). SUMOylation has been described to promote or disrupt protein–protein interaction making the influence of this modification on gene expression context dependent (27). Thus, a possible explanation for SUMOylation-driven Hsf1 activity attenuation could be that Hsf1 SUMOylation affects the interaction with the transcriptional machinery by modulating the interaction with positive transcription elongation factor b (23) or by recruiting transcriptional corepressors like histone deacetylases (17).

**Figure 8:**
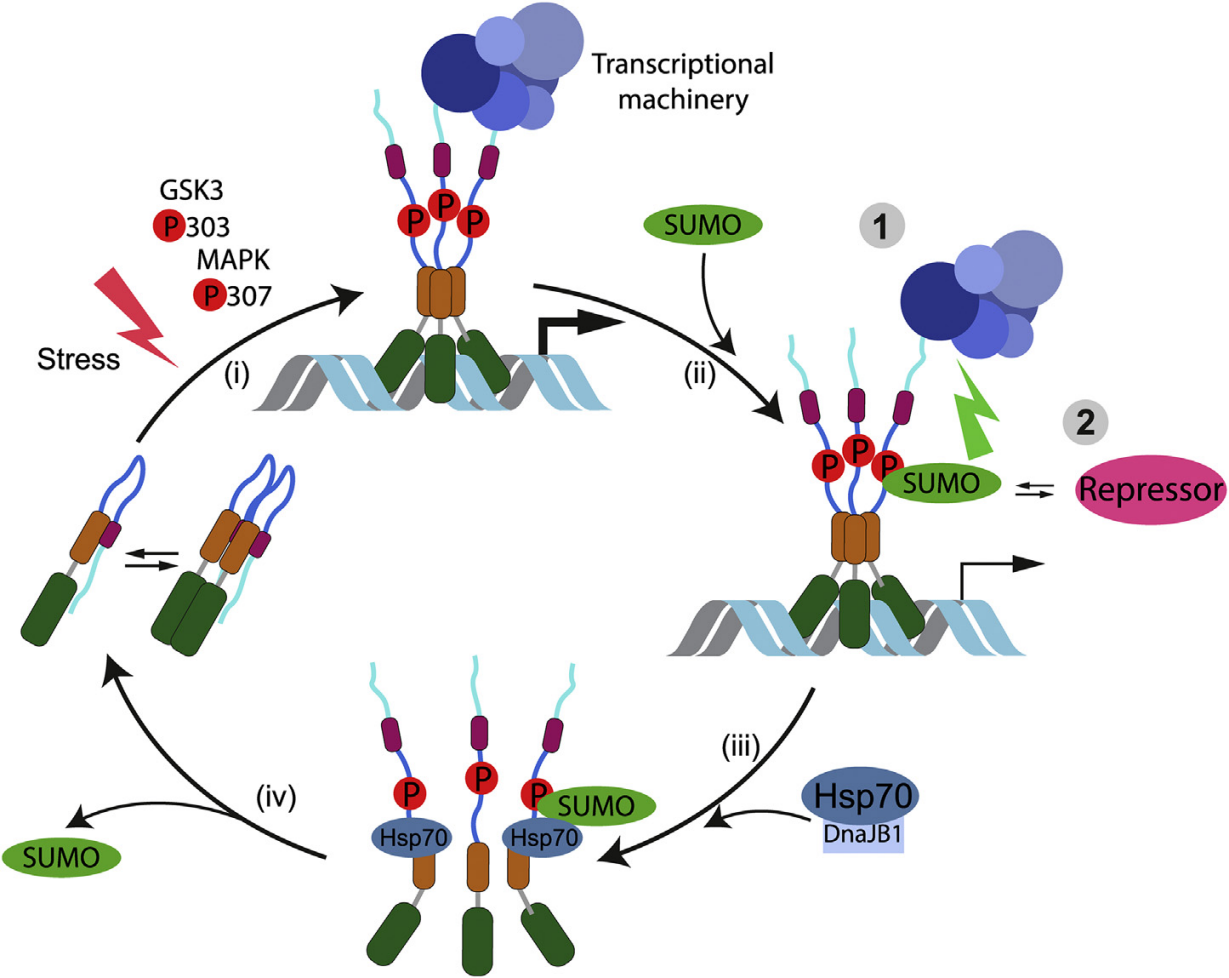
**Model of heat shock transcription factor 1 (Hsf1) activity regulation by SUMOylation.** (i) Hsf1 trimerizes upon stress. The activation is accompanied by Hsf1 phosphorylation. Among others, serine 307 is phosphorylated by mitogen-activated protein kinase (MAPK), and serine 303 is phosphorylated by glycogen synthase kinase 3 (GSK3). (ii) Upon transcriptional activation, Hsf1 is SUMOylated by the SUMOylation machinery, and its activity is repressed by 1: impairment of Hsf1 interaction with transcriptional machinery or/and 2: recruitment of corepressors. (iii) Hsf1 SUMOylation does not impair DNA binding or Hsp70-mediated dissociation from DNA. (iv) Hsf1 SUMOylation is transient. Hsf1 is recovered to a resting state by the Hsp70 system. SUMO, small ubiquitin-like modifier.

In summary, our data together with previous studies suggest a sequential Hsf1 regulation model where stress induces Hsf1 trimerization, mitogen-activated protein kinase–mediated phosphorylation at serine 307 and glycogen synthase kinase 3–mediated phosphorylation of serine 303. Both events, trimerization and phosphorylation, enhance Hsf1 SUMOylation efficiency. SUMO modification decreases Hsf1 activity by impairing interaction with activators of transcription or recruiting corepressors of transcription. Both, modified and unmodified, forms of Hsf1 can be dissociated from DNA by the Hsp70 system to attenuate the HSR (Fig. 8).

## Experimental procedures

### Protein production and purification

Human Hsf1 was purified as a His_6_–SUMO fusion. *Escherichia coli* BL21(DE3) Rosetta cells were freshly transformed with a plasmid encoding His_6_–SUMO–Hsf1. Overnight preculture was grown at 30 °C, and then, bacteria were grown at 37 °C to an absorbance of 0.6 at 600 nm. Temperature was shifted to 20 °C, and protein overproduction was induced by addition of 0.1 mM IPTG. The culture was grown for 2 h at 20 °C, and cells were subsequently harvested by centrifugation (5000*g*; 4 °C for 10 min). Bacterial pellet was resuspended in 20 ml of Hsf1 lysis buffer (25 mM Hepes/KOH pH 7.5, 100 mM KCl, 5 mM MgCl_2_, and 10% glycerol). Bacterial suspension was dropwise frozen in liquid nitrogen and stored at −80 °C. All following steps need to be carried out at 4 °C. Cells were disrupted using a Mixer Mill MM400 (Retsch) and resuspended in 200 ml Hsf1 lysis buffer supplemented with 3 mM β-mercaptoethanol, DNase, and protease inhibitors (10 μg/ml aprotinin, 10 μg/ml leupeptin, 8 μg/ml pepstatin, and 1 mM PMSF). The resulting lysate was centrifuged (33,000*g*; 4 °C for 45 min) to remove cell debris. The soluble fraction containing His_6_–SUMO–tagged Hsf1 was incubated for 25 min at 4 °C with 0.4 g Protino Ni–iminodiacetic acid resin (Macherey-Nagel) in a rotation shaker. The resin was transferred to a gravity-flow column and washed with 100 ml of Hsf1 lysis buffer. Bound protein was eluted with Hsf1 lysis buffer containing 250 mM imidazole, 3 mM β-mercaptoethanol, and protease inhibitors. The His_6_–SUMO tag was cleaved off by incubation with tobacco etch virus protease for 1 h at 4 °C. The cleaved Hsf1 was further separated by SEC on Superdex200 HiLoad 16/60 column (GE Healthcare), equilibrated with Hsf1 SEC buffer (25 mM Hepes/NaOH, pH 7.5, 150 mM NaCl, 10% glycerol, and 2 mM DTT). The fractions containing either monomeric or trimeric Hsf1 were concentrated to 10 μM, flash frozen in liquid nitrogen and stored at −80 °C.

After large-scale SUMOylation, the sample was subjected to SEC on a Superdex 200 10/300GL column (GE Healthcare), equilibrated with Hsf1 SEC buffer (25 mM Hepes/NaOH, pH 7.5, 150 mM NaCl, 10% glycerol, and 2 mM DTT).

Human Hsc70 (HSPA8) was purified as a His_6_–SUMO fusion from overproducing *E. coli* BL21(DE3) Rosetta cells, which were resuspended in Hsc70 lysis buffer (50 mM Tris, pH 7.5, 300 mM NaCl, 5 mM MgCl_2_, and saccharose 10%) supplemented with 3 mM β-mercaptoethanol, DNase, and protease inhibitors (10 μg/ml aprotinin, 10 μg/ml leupeptin, 8 μg/ml pepstatin, and 1 mM PMSF). Bacteria were lysed using a chilled microfluidizer (MicroFluidizer EmulsiFelx-C5; Avestin; C505113) at a pressure of 1000 bar. The resulting lysate was centrifuged (33,000*g*, 4 °C for 45 min), and the supernatant was incubated for 25 min at 4 °C with 2 g Protino Ni–iminodiacetic acid resin (Macherey-Nagel) in a rotation shaker. The resin was transferred to a gravity-flow column, washed with 200 ml of Hsc70 lysis buffer followed by high salt (Hsc70 lysis buffer but 1 M NaCl) and ATP (Hsc70 lysis buffer with 5 mM ATP) washes. Bound protein was eluted with Hsc70 lysis buffer containing 300 mM imidazole, 3 mM β-mercaptoethanol, and protease inhibitors. Fractions containing His_6_–SUMO–Hsc70 were desalted (HiPrep 26/10 Desalting column; GE Healthcare) to HKM150 buffer (25 mM Hepes/KOH, pH 7.6, 150 mM KCl, and 5 mM MgCl_2_), concentrated to 50 μM, aliquoted, flash frozen in liquid nitrogen, and stored at −80 °C.

Human DnaJB1/Hdj1 was purified without tag from *E. coli* BL21(DE3) Rosetta cells after overproduction for 4 h at 30 °C. Cells were resuspended in DnaJB1 lysis buffer (50 mM Tris/HCl, pH 8, 10 mM DTT, 0.6% [w/v] Brij 58, and 2 mM MgCl_2_) supplemented with DNase and 1 mM PMSF. Bacteria were lysed using chilled microfluidizer at a pressure of 1000 bar. The resulting lysate was centrifuged (33,000*g*; 4 °C for 45 min). One volume of buffer A (50 mM sodium phosphate buffer, pH 7, 5 mM DTT, 1 mM EDTA, and 0.1% [w/v] Brij 58) was added to the clarified lysate. DnaJB1 was then precipitated with 65% ammonium sulfate. The obtained precipitate was diluted in buffer B (50 mM sodium phosphate buffer, pH 7, 5 mM DTT, 1 mM EDTA, 0.1% [w/v] Brij 58, and 2 M urea) and dialyzed against buffer B. DnaJB1 was loaded onto a cation exchange resin (SP Sepharose) and eluted with a 0 to 666 mM KCl gradient in 15 CV. DnaJB1-containing fractions were combined, dialyzed against buffer C (50 mM Tris/HCl, pH 7.5, 2 M urea, 0.1% [w/v] Brij 58, 5 mM DTT, and 50 mM KCl) and subsequently loaded onto a hydroxyapatite resin. Bound protein was eluted with increasing concentration of buffer D (50 mM Tris/HCl, pH 7.5, 2 M urea, 0.1% [w/v] Brij 58, 5 mM DTT, 50 mM KCl, and 600 mM KH_2_PO_4_). DnaJB1-containing fractions were dialyzed against HKMG300 buffer (25 mM Hepes, pH 7.6, 5 mM MgCl_2_, 300 mM KCl, and 10% glycerol), aliquoted, flash frozen in liquid nitrogen, and stored at −80 °C.

### Comments

Please note, the nomenclature for mammalian SUMO2 and SUMO3 is used inconsistently. Like many colleagues in the SUMO field, we follow the nomenclature as introduced by Saitoh and Hinchey (22). Their assignment was consistent with the original description of mammalian SUMO genes (reviewed in (50)). According to this, mature SUMO2 (Smt3A) is 92 amino acids long, mature SUMO3 (Smt3B) consists of 93 amino acids.

### Purification of SUMOylation machinery components

Unless stated otherwise, protein purification protocols involved IPTG-induced expression in *E. coli* BL21 gold (Stratagene), bacterial lysis with lysozyme, and a 100,000*g* spin for 1 h to collect soluble proteins (see also Ref. (51)). Each buffer contained 1 μg/ml each of leupeptin, pepstatin, and aprotinin, and 1 mM DTT (or β-mercaptoethanol); lysis buffers also contained 0.1 mM PMSF. After the specific purification steps described later, proteins were aliquoted, flash frozen, and stored at −80 °C. The final buffer in each protocol was transport buffer (20 mM Hepes, 110 mM potassium acetate, 2 mM magnesium acetate, and 0.5 mM EGTA).

#### Human E1 enzyme

Purification involved coexpression of His–Aos1 and Uba2, bacterial lysis in 50 mM sodium phosphate (pH 8), 300 mM NaCl, 10 mM imidazol, purification on ProBond Resin (Invitrogen), and SEC on Superdex200 HiLoad 16/60 column (GE Healthcare) and ion exchange chromatography (Mono Q; Pharmacia Biotech).

#### Human E2 enzyme (Ubc9)

Purification involved lysis in 50 mM sodium phosphate (pH 6.5), 50 mM NaCl, incubation of the 100,000*g* supernatant with SP-sepharose beads (SIGMA), elution of Ubc9 from the beads with 50 mM sodium phosphate (pH 6.5), 300 mM NaCl, and SEC on Superdex200 HiLoad 16/60 column (GE Healthcare).

#### Human E3 ligases

PIAS proteins were expressed in Rosetta 2 cells transformed with pGEX–6P–PIAS1–His, pGEX4T1 PIAS3, or pGEX4T1–PIAS4 in autoinducing medium ZYM-5052 for 48 h at 16 to 18 °C. The cells were lysed in 50 mM Tris, pH 8.0, 300 mM NaCl, 10% glycerin, 50 μM ZnCl_2_, 0.2% Triton X-100, 1 μg/ml of each aprotinin, leupeptin, pepstatin, 1 mM pefabloc, and 1 mM DTT. Lysates were cleared by centrifugation at 100,000*g* and incubated with GSH agarose. Bound proteins were eluted with 30 mM glutathione and separated over a Superdex200 10/300 column in 50 mM Tris, pH 8.0, 200 mM NaCl, 50 μM ZnCl_2_, 1 μg/ml each of aprotinin, leupeptin, pepstatin, and 1 mM DTT. GST–IR1+M was expressed in BL21(DE3) from pGEX–3X–RanBP2–IR1+M (RanBP2 aa 2631–2711), purified and cleaved with factor Xa as described in Ref. (51). The final buffer after gel filtration on Superdex 75 was transport buffer (20 mM Hepes, 110 mM potassium acetate, 2 mM magnesium acetate, and 0.5 mM EGTA).

#### Human SUMO1/SUMO2/HisSUMO1

Purification involved bacterial lysis in 50 mM Tris/HCl (pH 8), 50 mM NaCl by sonification, preclearing of the 100,000*g* supernatant with Q sepharose (SIGMA) in case of untagged SUMO (in case of HisSUMO1, nickel affinity chromatography was performed), concentration, and subsequent SEC on Superdex75 HiLoad 16/60 column (GE Healthcare).

### *In vitro* SUMOylation assays

#### Comparison of SUMOylation yield *in vitro*

Hsf1 *in vitro* SUMOylation reactions were set up to 20 μl in the assay buffer (20 mM Hepes/KOH, pH 7.3, 110 mM KAcO, 2 mM Mg(AcO)_2_, 1 mM EGTA, 0.05% Tween, 0.2 mg/ml ovalbumin, 1 mM DTT, and 1 μg/ml of each aprotinin, leupeptin, and pepstatin) and consisted of 0.1 μM SUMO E1 (His–Aos1/Uba2), 0.25 μM or 1.1 μM (high) SUMO E2 (untagged Ubc9), E3 ligase (if applied): 50 nM IR1+M (fragment of RanBP2; GST–IR1+M was used for Fig. 1*C*), 50 nM GST–PIAS1, 24 nM GST–PIAS3, 50 nM GST–PIAS4, 10 μM His–SUMO1 or 9 μM SUMO1/SUMO2 (untagged), 0.5 μM of monomeric or trimeric (purified as a trimer or monomer heat shocked) Hsf1 (wt, S303E/S307E, K298R, S303E/S307E/K298R), and 5 mM ATP. After ATP addition, reactions were incubated for 1 or 3 h at 25 °C. Samples were separated by SDS-PAGE, blotted onto a polyvinylidene difluoride (PVDF) membrane, and subsequently detected with an Hsf1-specific antibody.

### Mass specrometry

The indicated fraction of the SEC separation (Superdex 200 10/300) of a SUMOylation reaction (20 pmol of protein mixture; 0.1 μM, 200 μl) was acidified by adding formic acid (FA) to 0.3% final concentration and loaded onto the trap column (POROS 10R1; Applied Biosystems) of a liquid chromatography system coupled to a MaXis electrospray ionization–quadrupole time-of-flight mass spectrometer (Bruker). The sample was desalted with 0.3% FA in MS grade water for 3 min and eluted with isopropanol/water (80/20, v/v) with 0.3% FA into the mass spectrometer. To calculate the molecular weight of the proteins present in the sample, the acquired mass spectra were deconvoluted using the maximum entropy algorithm provided by the Data Analysis software of the mass spectrometer.

### Fluorescence polarization assays

#### Preparation of fluorescently labeled DNA (ds-Alexa488–HSEs)

Fluorescently labeled ds-Alexa488–HSEs were prepared by annealing of fluorescently labeled Alexa488–3xHSE sense oligonucleotides (5’-[A488]-ccccTTCccGAAtaTTCcccc) with 3xHSE antisense nucleotides (5’-ggggGAAtaTTCggGAAgggg) (2 min at 70 °C, 0.6 °C/min stepwise decrease from 70 to 30 °C).

#### SUMOylation on DNA-bound Hsf1

*In vitro* SUMOylation reactions were set up to 20 μl in the assay buffer (25 mM Hepes, pH 7.6, 150 mM KCl, 5 mM MgCl_2_, 10% glycerol, and 1 mM DTT) and consisted of 0.1 μM SUMO E1 (His–Aos1/Uba2), 1.1 μM SUMO E2 (untagged Ubc9), 10 μM SUMO2 (untagged), 5 mM ATP, and 0.3 μM trimeric Hsf1 (S303/307E variant) bound to DNA (25 nM ds-Alexa488–HSEs). After preparation of samples, the plate was spun down at 1000*g* for 1 min at RT. Fluorescence anisotropy of the prepared samples was measured at 25 °C for 2 h using a plate reader (CLARIOstar; BMG Labtech; excitation, F:482-16 and emission, F:530-40). Samples were separated by SDS-PAGE, blotted onto a PVDF membrane, and subsequently detected with an Hsf1-specific monoclonal antibody.

#### Binding of trimeric Hsf1 to HSEs

About 5 nM ds-Alexa488–HSEs were titrated with trimeric Hsf1 at different concentrations ranging from 0.16 to 166.67 nM on low-volume 384-well plate (CORNING; REF3820). SUMOylation was carried out at 30 °C for 2 h (assay buffer: 25 mM Hepes, pH 7.6, 150 mM KCl, 5 mM MgCl_2_, 10% glycerol, 1 mM DTT, E1/E2/S2/Hsf1 equaled 0.08/0.92/8.33/1 μM, and 4.17 mM ATP). The plate was subsequently spun down at 1000*g* for 1 min at RT. Fluorescence anisotropy of the prepared samples was measured after 15 min at 25 °C using a plate reader (CLARIOstar; BMG Labtech; excitation, F:482-16 and emission, F:530-40). The data points were fitted to the quadratic solution of the law of mass action using the GraphPad Prism software (GraphPad Software).

#### Hsf1 dissociation from HSEs

About 7.5 μM SUMO–Hsc70 and 2 mM ATP in HKMG150 buffer (25 mM Hepes, pH 7.6, 150 mM KCl, 5 mM MgCl_2_, 10% glycerol, and 2 mM DTT) preincubated at 25 °C for 30 min were mixed with 10 μM Hdj1/DNAJB1, 2 mM ATP, 100 nM trimeric Hsf1, and 25 nM HSEs preincubated at 25 °C for 5 min on low-volume 384-well plate (CORNING; REF3820) in a final 20 μl reaction volume (modified from Ref. (34)). The plate was subsequently spun down at 1000*g* for 1 min at RT. Fluorescence anisotropy of the prepared samples was monitored in a plate reader (CLARIOstar; BMG Labtech; excitation, F:482-16 and emission, F:530-40) at 25 °C. A trimeric Hsf1 fraction was used for the experiments, if not stated otherwise in the legend to the figures. Data were normalized to samples where Hsf1 was absent. A single exponential equation with dissociation delay was fitted to the data points using the GraphPad Prism software:

$$y=y_{max}\;for\;t\leq t_{0}\;\text{and}$$

$$y=y_{0}+\left(y_{max}-y_{0}\right)*e^{-k\left(t-t_{0}\right)}\;for\;t>t_{0}$$

with *y*_*max*_ and *y*_0_ representing the fitted maximal and minimal FP values and k being the rate of the dissociation reaction.

#### SDS-PAGE

Protein samples premixed with 5× SDS-PAGE sample buffer (250 mM Tris, pH 6.8, 50% glycerol, 10% SDS, 25% β-mercaptoethanol, and 0.5% bromophenol blue) were separated on 12% SDS-PAGE gels in the Laemmli system (running buffer: 25 mM Tris, 192 mM glycine, and 0.1% SDS). Separation was carried out at constant current (50 mA per gel; 28 min). Gels were stained using a commercial staining solution (Quick Coomassie Stain, SERVA) or used further for Western blot analysis.

#### BN-PAGE

Protein samples premixed with 4× BN-PAGE sample buffer (250 mM Tris, pH 6.8, 40% glycerol, and 0.1% Coomassie Brilliant Blue G 250) were separated on 8% PAGE gels in Laemmli system (cold BN-PAGE running buffer: 25 mM Tris and 0.2 M glycine). Separation was carried out at constant current (20 mA per gel, 1 h, on ice). Gels were subsequently stained using staining solution (Quick Coomassie Stain; Serva) or used further for Western blot analysis.

#### Western blot α-Hsf1

Proteins separated on PAGE gel were transferred to a PVDF membrane (Immobilon-P/Immobilon-FL; 0.45 μm; Merck Millipore) using Trans-Blot Turbo Transfer System (Bio-Rad). The membrane was subsequently blocked in PBS-T buffer (137 mM NaCl, 2.7 mM KCl, 10 mM Na_2_HPO_4_, 1.8 mM KH_2_PO_4_, and 0.05% Tween 20) containing 1% milk for 45 min at RT. After blocking, the membrane was incubated with primary antibody (HSF1 [H-311] rabbit polyclonal IgG or HSF1 [E-4] mouse monoclonal IgG; Santa Cruz Biotechnology; 1:1000 dilution) overnight at 4 °C on a roller. The next day, the membrane was washed 3 times for 30 min with PBS-T buffer and incubated with the secondary fluorescently labeled antibody (Goat anti Rabbit IgG IRDye 680RD; Odyssay, 1:20,000; Goat anti-Mouse IRDye 800CW; Odyssay, 1:20,000; Donkey anti-Rabbit IgG [H + L] horseradish peroxidase conjugated; Jackson ImmunoResearch, 1:10,000). After 1 h of incubation, the membrane was washed 3 times for 30 min with PBS-T buffer. The protein of interest was further detected using fluorescence (LICOR Odyssey Infrared Imaging System; 700 nm or 800 nm channel). Western blots were quantified using Image Studio Lite, version 5.2 (LI-COR Biosciences GmbH).

### Quantification and statistical analysis

All biochemical assays were performed at least 3 times independently. Data were analyzed with GraphPad Prism 6.0 software. Statistical significance was estimated by ANOVA or *t* tests as indicated in the legends to the figures. For quantification, ImageJ or Image Studio Lite, version 5.2, was applied.

## Data availability

All the data are contained within the article.

## Conflict of interest

The authors declare that they have no conflicts of interest with the contents of this article.
