# Supplementary material for: Heat shock transcription factor 1 is SUMOylated in the activated trimeric state
Source: J Biol Chem. 2021 Jan 23;296:100324. doi: 10.1016/j.jbc.2021.100324 (PMC7949154; doi:10.1016/j.jbc.2021.100324)
Supplement: Figure S1 [file mmc1.pdf]

Heat shock transcription factor 1 is SUMOylated in the activated trimeric state

Szymon W. Kmiecik, Katarzyna Drzewicka, Frauke Melchior and Matthias P. Mayer

Supplemental information

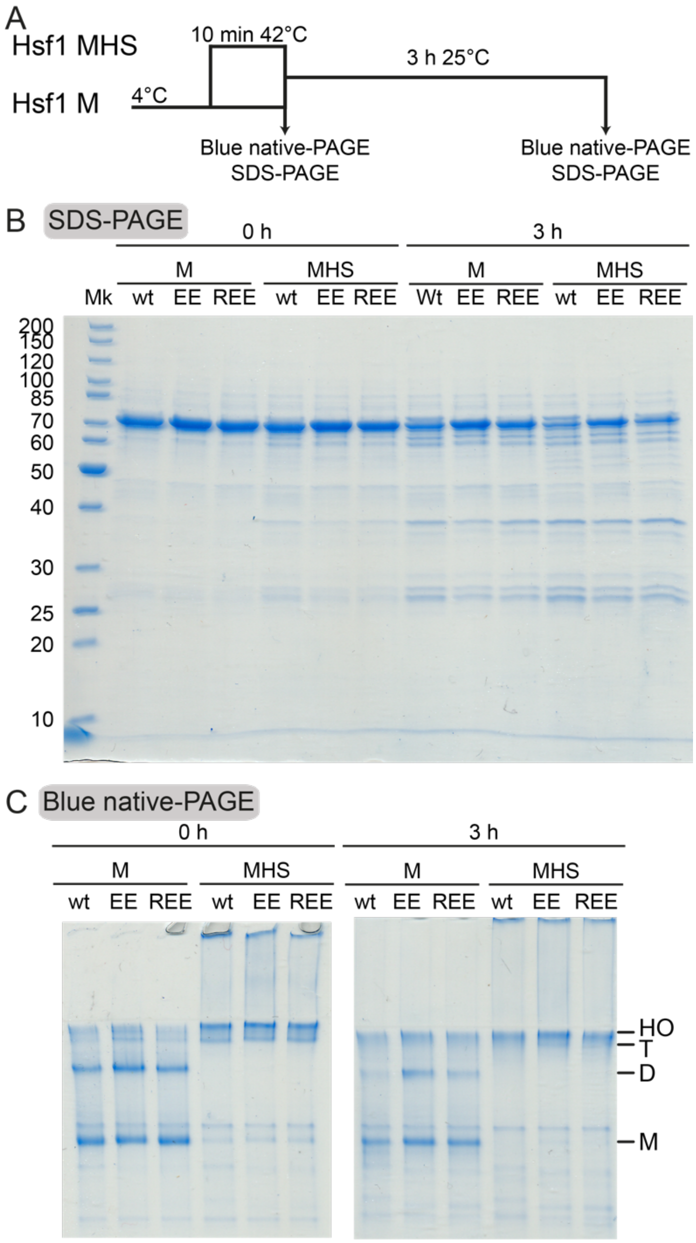

Supplemental Figure S1: Oligomeric state of Hsf1 does not change during incubation time.

A, Scheme of the experiment: monomeric wild type Hsf1 (wt), Hsf1-S303E,S307E (EE), and Hsf1-K298R,S303E,S307E (REE) were either kept at 4°C for 10 min (M) or trimerized by incubation at 42°C for 10 min (MHS) and subsequently incubated at 25°C for 3 h.

B-C, Samples after 10 min pre-incubation (0 h) and after 3 h were analyzed by SDS-PAGE (**B**) and blue-native PAGE (**C**) with subsequent Coomassie Brilliant Blue staining. HO, higher order oligomers; T, trimers; D, dimers; M, monomers.
